# Inferring Diagnostic and Prognostic Gene Expression Signatures Across WHO Glioma Classifications: A Network-Based Approach

**DOI:** 10.1177/11779322241271535

**Published:** 2024-09-15

**Authors:** Roberta Coletti, Mónica Leiria de Mendonça, Susana Vinga, Marta B. Lopes

**Affiliations:** 1 Center for Mathematics and Applications (NOVA Math), NOVA FCT, NOVA University of Lisbon, Caparica, Portugal; 2 INESC-ID, Instituto Superior Técnico, Universidade de Lisboa, Lisbon, Portugal; 3 IDMEC, Instituto Superior Técnico, Universidade de Lisboa, Lisbon, Portugal; 4 NOVA School of Science and Technology (NOVA FCT), NOVA University of Lisbon, Caparica, Portugal; 5 UNIDEMI, Department of Mechanical and Industrial Engineering, NOVA FCT, NOVA University of Lisbon, Caparica, Portugal

**Keywords:** graphical lasso, regularized cox regression, survival analysis, RNA-Seq, glioma, TCGA, WHO CNS classification, networks, variable selection, biomarkers

## Abstract

Tumor heterogeneity is a challenge to designing effective and targeted therapies. Glioma-type identification depends on specific molecular and histological features, which are defined by the official World Health Organization (WHO) classification of the central nervous system (CNS). These guidelines are constantly updated to support the diagnosis process, which affects all the successive clinical decisions. In this context, the search for new potential diagnostic and prognostic targets, characteristic of each glioma type, is crucial to support the development of novel therapies. Based on The Cancer Genome Atlas (TCGA) glioma RNA-sequencing data set updated according to the 2016 and 2021 WHO guidelines, we proposed a 2-step variable selection approach for biomarker discovery. Our framework encompasses the graphical lasso algorithm to estimate sparse networks of genes carrying diagnostic information. These networks are then used as input for regularized Cox survival regression model, allowing the identification of a smaller subset of genes with prognostic value. In each step, the results derived from the 2016 and 2021 classes were discussed and compared. For both WHO glioma classifications, our analysis identifies potential biomarkers, characteristic of each glioma type. Yet, better results were obtained for the WHO CNS classification in 2021, thereby supporting recent efforts to include molecular data on glioma classification.

## Introduction

Glioma is one of the most common brain tumors, which in adults represents more than 80% of malignant cancers in this organ.^1^ One of the reasons why gliomas often have a bad prognosis is because of their high heterogeneity, which arises from differences affecting histological and molecular levels.^2^ Glioma samples are characterized by different molecular profiles and tumor cell types, which result in distinct glioma types. The criteria for glioma classification are constantly revised according to new discoveries. For many years, the diagnosis of glioma has been exclusively based on histology, being recently subjected to a substantial revision with the inclusion of genetic information in the classification procedure. Despite the importance of this continuous update, it represents a challenge for diagnosis and the design of effective therapies.^3^

The World Health Organization (WHO) classification of the central nervous system (CNS) introduced molecular characteristics as part of the diagnosis of glioma tumors in 2016.^4^ This novelty represented a valuable improvement, given the subjectivity of the histological evaluation.^5^ However, with this integrated diagnosis, contradictory results could arise, in case of patients showing inconsistent histological and molecular features.^4^

In 2021, the glioma classification procedure was updated,^6^ mainly focusing the diagnosis on the evaluation of objective genetic features and solving the problem of possible contradictory diagnostic results that could arise from the 2016 approach. In the latest guidelines release, the status of the isozymes of the isocitrate dehydrogenase (IDH) gene family and the combined loss of the short arm chromosome 1 and the long arm of chromosome 19 (1p/19q codeletion), already introduced in 2016, became central for the classification of glioma types. In particular, IDH-mutant samples are classified as oligodendroglioma if presenting 1p/19q codeletion; conversely, the diagnosis of astrocytoma is assigned. The IDH-wild-type samples are classified as glioblastoma (GBM) if certain histological features or genetic parameters are present. These modifications in the glioma classification as defined by the WHO CNS guidelines aimed to support as best as possible the diagnosis, which is the first essential step to designing an effective therapy.^7^ Indeed, given the deep differences in glioma, each type could be influenced by specific markers, having an impact on cancer development.^8^ In this context, discovering the existing underlying characteristics could be crucial to improve patient prognosis.^3^

Nowadays, thanks to modern data extraction techniques and gene sequencing advances, large sets of data are available. The huge dimension of these data sets, as well as the hidden relations existing between biological entities, makes it challenging to infer information linking variables and clinical outcomes. To this end, mathematical and statistical methods can be employed to tackle the problem and to identify potential therapeutic targets.

The high dimensionality of the data poses challenging problems in model identification and parameter estimation, which can be addressed by means of regularization techniques. These methods consist of adding a penalty term to the cost function of the chosen statistical model. In recent years, regularization has been widely used in bioinformatics applications, and several methods have been developed to address this need.^9^ However, these classical regularization methods usually perform univariate variable selection, which is unfeasible when considering entities, such as genes, interacting in a complex network. In this context, graph theory can effectively be employed to infer the underlying network structure, being a valuable approach used in precision oncology,^10,11^ and in glioma.^12^ Among the many techniques developed to estimate a graph starting from real data,^13^ in this work, we employed graphical lasso, a widely known method combining regularization and network estimation.^14^ Graphical lasso estimates relations between variables introducing sparsity through lasso regularization. It is particularly accurate for huge data sets with number of features (p) much larger than the number of sample (n), and it has been applied in many fields.^15-17^

Besides the identification of meaningful diagnostic features from the overall network, the evaluation of their prognostic role is an invaluable contribution. Regularized survival analysis can be used to this end. Here, we used the Cox regression model, which is able to determine the association of a set of features with the time until the event of interest occurs.^18^ Coupling survival analysis with regularization techniques allows for the selection of features carrying information about the overall network and survival, and, therefore, promising as prognostic biomarkers.^9^

In this work, we considered data from The Cancer Genome Atlas (TCGA) program, consisting of a huge data set (p≫n) of RNA-sequencing (RNA-Seq), from the LGG (lower-grade glioma, grouping both astrocytoma and oligodendroglioma types) and GBM projects.^19-21^ These data sets have been preprocessed to assess the graphical lasso hypothesis and to update the sample classification according to the 2016 and the 2021 WHO CNS guidelines.^22^ Through the graphical lasso method, we reduced the dimensionality of the data set by selecting a subset of variables for each glioma type, and we analyzed the results by identifying key features (*hubs*) within the estimated networks. We performed survival analysis through Cox regression model^18^ with regularization with the lasso penalty,^23^ by considering (1) the set of selected variables, (2) the variables that have been exclusively selected from each glioma type, and (3) the hub genes. This approach allowed us to assess whether the variables selected for their diagnostic relevance also carry prognostic information.

The article is structured as follows. In the “Materials and Methods” section, we start introducing the underlying theory of graphical lasso and of Cox regression modeling with lasso regularization, and we describe the data sets preprocessing and workflow analysis. In the “Results” section, the model outcomes are presented and analyzed, followed by a comprehensive discussion of the results, with correspondent biological interpretation. Finally, the conclusion resumes our main contribution.

## Material and Methods

### Graphical lasso

Let G be a graph with $X=(X_{1},\ldots ,X_{p})$ nodes (variables) having a multivariate normal distribution $X∼N_{p}(0,\Sigma ).$ Let Σ and S be the theoretical and empirical covariance matrix, respectively.

The graphical lasso,^14^ herein designated as glasso, finds the precision matrix $\Theta =\Sigma^{-1},$ by solving the following Gaussian log-likelihood maximization problem:

(1) $$\underset{\Theta }{\max}\{\log(\det\Theta )-tr(S\Theta )-\rho ||\Theta {\left|\right|}_{1}\},$$

where tr(⋅) is the trace operator. The presence of the regularization term $\rho ||\Theta {\left|\right|}_{1}$ serves as penalty, providing a weight on the elements of the matrix Θ, and inducing sparsity into the solution. The value of the regularization parameter ρ determines the degree of sparsity. It has been proved that the network structure of the graph G can be estimated based on the nonzero entrances of Θ. ^24^ In particular, if $\Theta_{i,j}=0,$ for certain i and j (i≠j), it means that the variables $X_{i}$ and $X_{j}$ are conditionally independent given the others $X_{k},\;k=1,\ldots ,n,\;k\neq i,j.$

### Cox regression modeling with lasso regularization

Cox regression is a survival model that relates the rate of an event happening in a given time point (eg, death) with several factors.^18^ Given p features (eg, gene expressions) for n samples, and let $(Y_{i},\;\delta_{i}),$ i=1,…,n be, respectively, the survival time and the indicator of the occurrence of the event $\left(\delta_{i}\in \{0,\;1\}\right)$ of the *i*^th^ patient. The goal of a survival models is estimating the hazard function, ie, the failure rate depending on time. If we define that function as:

$$h(t|X^{i})=h_{0}(t)\exp X^{i}\beta ,$$

where $h_{o}(t)$ is a baseline hazard function, the Cox regression model determines the coefficients $\beta =(\beta_{1},\ldots ,\beta_{p})$ that maximize the following partial log-likelihood function:

(2) $$\underset{\beta }{\max}\sum_{i=1}^{n}\delta_{i}\left[X^{i}\beta -\log\left(\sum_{j:Y_{j}\geq Y_{i}}X^{j}\beta \right)\right],$$

where $X^{i}=\left(X_{1}^{i},\ldots ,X_{p}^{i}\right)$ is the set of features of the *i*^th^ patient.

To study the relationship between predictor variables and survival outcome in a high-dimensional scenario (p≫n), the Cox regression model can be regularized by including a penalty function P(β). In this work, we considered lasso regularization;^23^ therefore, $P(\beta )=\lambda ||\beta {\left|\right|}_{1}.$

### Data set preprocessing and framework

**Figure 1.**
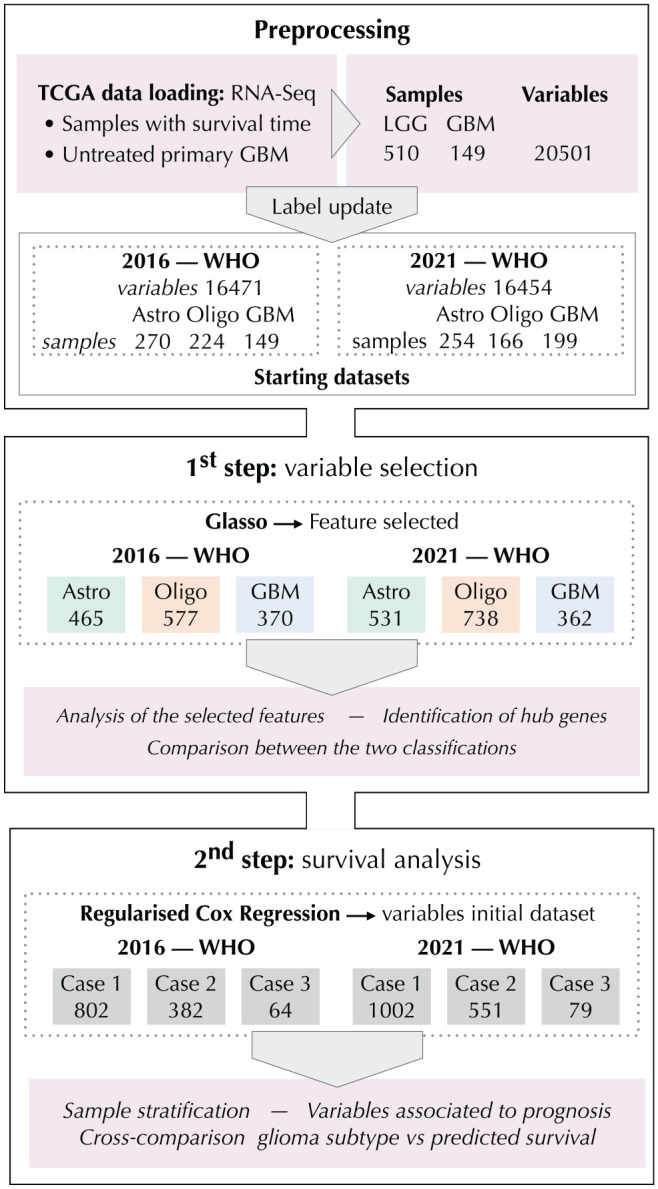
Workflow of the analysis. After the preprocessing of the TCGA-LGG and TCGA-GBM databases, we obtained 6 data sets (3 for each classification procedure, 2016 WHO and 2021 WHO). First step: Glasso algorithm has been applied separately on each glioma type, obtaining the network-based variable reduction. The number of features selected for any given type is reported in the second box of the pipeline. Green indicates astrocytoma (Astro), orange is oligodendroglioma (Oligo), and blue represents GBM. Second step: The features selected by the previous step are used as input for the survival analysis. All the data sets consider a matrix comprising all the glioma samples and different variables, depending on the case of study. *Case 1* takes into account all the selected variables. *Case 2* refers to the variables exclusively selected from each glioma type. *Case 3* considers only the hub genes. Boxes in figure indicate the number of variables of the starting data sets. In each step, the analysis and the comparison between the 2 classification outcomes have been performed.

The data set selection procedure and the workflow of our analysis are summarized in Figure 1. The glioma data set we used in this study is available on TCGA portal, under the LGG and GBM projects, and it has been downloaded by the getFirehoseData function of **RTCGAToolbox** package,^25^ from R software (version 3.5.1, https://www.r-project.org). This data set contains expression levels from 20 501 genes, normalized by transcript per million and quartile normalizations. We updated the patients’ diagnosis according to both 2016 and 2021 WHO CNS guidelines, by integrating patient’ molecular profiles^26^ and reproducing the procedure explained by de Mendonça et al.^22^ We considered samples reporting the survival time information, to perform survival analysis. Regarding the GBM data set, we considered samples related to “untreated primary gbm.”

To assess the assumption of normality required by glasso, we applied the normalization with huge.npn function from the **huge** R package,^27^ which implements the gausianization through the nonparanormal transformation. For our study, we considered only the variables with normal distribution, according to the Jarque-Bera test.^28^ Finally, following the 2016 WHO CNS classification, our reference data set contains 16 471 normally distributed variables for 270 astrocytoma, 224 oligodendroglioma, and 149 GBM samples. Following the 2021 WHO classification, the data set is constituted of 16 454 normally distributed variables for 254 astrocytoma, 166 oligodendroglioma, and 199 GBM samples (Figure 1; preprocessing).

For the first step of our methodology, glasso has been applied to each data set, separately. The estimation of sparse networks led to a network-based variable selection, which has been mathematically validated to assess the reliability of the set of identified variables (see Supplemental Section S1). The process of variable selection produced a different subset of genes for each glioma type, namely 465, 577, and 370 genes for astrocytoma, oligodendroglioma, and GBM, considering 2016 WHO classification, whereas 531, 738, and 362 genes for astrocytoma, oligodendroglioma, and GBM, considering 2021 WHO classification (Figure 1; first step). These have been used as starting data sets to perform the second step of survival analysis, as explained in the following sections.

### Implementation of variable selection

The variable selection was performed by applying glasso through the huge.glasso function of the **huge** R package,^27^ by setting the regularization parameter ρ=0.9. This value has been empirically chosen to consistently reduce the number of variables and increase interpretability. Indeed, as mentioned in the “Graphical lasso” section, the regularization term determines the sparsity of the precision matrix Θ. As a consequence, setting a high value of ρ, many variables will result unconnected within the graph, allowing for a network-based variable selection. To identify key nodes in each network, we introduced 2 different measures.

Given a variable $X_{i},$ the *weight* measure consists in evaluating the degree of the *i*^th^ node as:

$$m_{i}=\sum_{j=1,j\neq i}^{n}|\theta_{ij}|,$$

whereas its *count* measure represents the number of the connection of the variable:

$$m_{i}=\sum_{j=1,j\neq i}^{n}q_{ij},$$

where $q_{i}=1,$ if $|\theta_{ij}|>0,$ $q_{i}=0$ otherwise.

As such, the genes can be sorted in ascending order according to both these measures, obtaining, for each data set, 2 ranked lists of genes. To identify the subset of the most relevant variables, for each list, we re-scaled the computed measures, by distributing them uniformly in [0,100]. Then, let $p_{i}$ be the value of the measure $m_{i}$ converted in percentage, we defined $X_{i}$ as a *hub* variable if $p_{i}>t,$ for a certain threshold t. The threshold parameter choice is a crucial point of our analysis. Setting too low values could result in big sets of variables, which is hard to interpret. Conversely, too high thresholds could remove important variables, which will be omitted from our analysis. The dimension of the set of variables selected through glasso also influences this choice, since the larger the set is, the more we need to filter our results. We manually analyzed the glasso results, with the aim of identifying a discriminating value able to divide the list of variables into the hubs and nonhubs groups. We observed that in astrocytoma and oligodendroglioma cases, the ranked list of genes reported very close percentage measures, ie, the difference between the measures of 2 following variables was almost constant, leading to no obvious choice for the threshold. On the contrary, the variables selected from GBM data set can clearly be divided into 2 groups, with a consistent variation of measure values around 60%. For this reason, we set t=60. Each measure selects a subset of hub features; thus, we considered as final hubs the union of these 2 subsets.

### Regularized Cox regression modeling, patient stratification, and survival analysis

Survival analysis has been performed by a regularized Cox regression model and implemented through the glmnet R package,^29^ by considering 3 cases. We considered a Pan-Glioma data set, composed of all glioma samples (LGG + GBM), and 3 cases are as follows: (1) all the variables selected by glasso, (2) the variables exclusively selected by each glioma type, and (3) only the features identified as hubs. This results in 6 data sets, ie, 3 cases for each glioma classification procedure (Figure 1—second step).

The parameter λ of lasso penalization during model fitting was determined by pmax, a parameter limiting the maximum number of candidate features to be selected to avoid overfitting. For this study, we have set pmax based on the 10 EPV (events per variable) rule of thumb,^30-32^ which relates the number of events with the maximum number of predictors that can be studied. Therefore, pmax was set as the ceiling function of the number of patients with the outcome event (death) divided by 10. The 2016 and 2021 WHO CNS classifications led to data sets composed of 233 and 226 events, which resulted in *pmax* = 24 and *pmax* = 23, respectively. Once defined pmax, the value of the parameter λ has been computed through the function glmnet, which determined the appropriate regularization parameter leading to the chosen number of variables. The data set dimensions and the parameters chosen in each case are summarized in Supplemental Table S2.

The function separate2GroupsCox from the glmSparseNet R package^33^ was used to separate the data into 2 groups of patients: high risk (HR) and low risk (LR). This function divides patients according to their prognostic index (PI), resulting from the matrix multiplication of the data with the fitted coefficients. By default, patients are ordered and stratified according to the median value of the PI distribution, ie, a patient is attributed to the LR group if its corresponding PI (computed using Cox model) is below or equal to the median risk, and assigned to the HR group otherwise. However, in all the defined data sets, the PIs exhibited a bivariate normal distribution, not centered by the median value (Supplemental Figure S1). Consequently, considering the default stratification is not suitable for our study, since it could force samples to belong to the wrong group.

To determine the threshold value in this imbalanced class situation, we computed a kernel density estimation of the distribution of the PI by the density R function, and we extracted the local minimum of such distribution between the 2 Gaussian peaks $(\hat{PI}).$ Samples with $\mathrm{PI}\leq \hat{PI}$ were assigned to the LR group, whereas samples having $\mathrm{PI}>\hat{PI}$ were considered to be part of the HR group. Supplemental Figure S1 shows the distribution of PIs and the corresponding threshold, in each case.

Based on this patient’ stratification, Kaplan-Meier survival curves were drawn and statistically compared with the log-rank test (hypothesis test to compare the distribution of time until the occurrence of an event in independent groups). A significance level of 0.05 was considered.

## Results

In this section, the results obtained from variable selection and survival analysis are shown. We discuss the outcomes in light of the comparison between the 2016 and 2021 WHO CNS guidelines, highlighting differences and similarities arising from the transcriptomics data. Finally, we used the obtained results as baseline knowledge to explore the 2021 estimated networks.

### Variable selection

**Figure 2.**
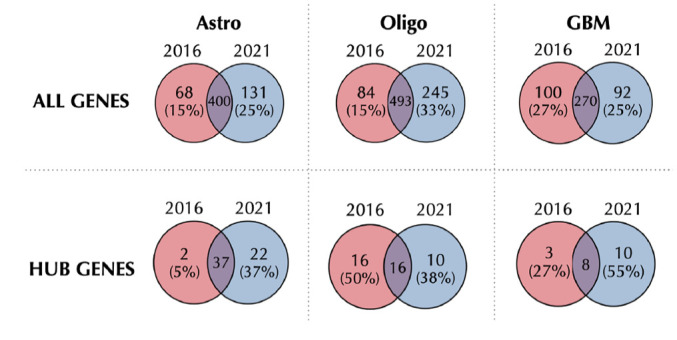
Venn diagrams showing the number of variables selected by glasso considering 2016 versus 2021 WHO CNS classification guidelines, for each glioma type (astrocytoma—Astro, oligodendroglioma—Oligo, and GBM). Percentages in brackets refer to the relative percentage of the exclusively selected variables for each set. The first row considers all the selected genes, whereas the second row is only about hub genes.

We performed the variable selection by considering each glioma type, separately, taking into account 2 classification procedures.^22^ We compared the genes which have been selected for a given tumor type, classified according to the 2 different guidelines (Figure 2; first row). For astrocytoma and oligodendroglioma, the number of variables selected by considering the 2021 WHO CNS classification was larger than the one obtained by following the 2016 guidelines. On the contrary, looking at the output derived from the 2016 classification, the percentage of variables exclusively selected for GBM is double the one of LGG (astrocytoma and oligodendroglioma). However, for all the types, the ranking of the genes that have been exclusively selected is very low, considering both weight and count measures, and, among these genes, there are no variables identified as hubs. The analysis has been repeated by considering the hub gene subsets to discuss the differences in selecting important features. Second row in Figure 2 shows the result of this comparison. For astrocytoma and GBM, most 2016 hub genes are identified as important also according to the 2021 WHO CNS sample classification, ie, 95% and 73% of astrocytoma and GBM 2016 hubs, respectively. In both cases, the 2021 classification led to a larger set of key features compared with 2016. Differently, for oligodendroglioma, our procedure selects more hub genes by starting from the data set obtained following the 2016 WHO CNS guidelines, with almost 50% of key genes shared by both classifications. Overall, all the hub genes (exclusive hubs included) are in the subset of selected variables of the corresponding glioma type, regardless of the classification procedure.

**Figure 3.**
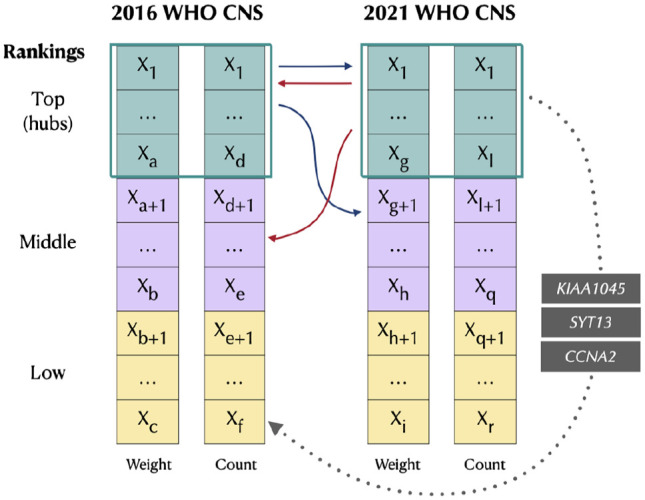
Cross-comparison of the hub gene rankings with respect to the other classification. Green, purple, and yellow vector sections represent top, middle, and low positions, respectively. Continuous arrows indicate the positions of most of the hubs. Dotted arrows highlight the 3 2021 hub genes having low 2016 rankings.

Given the genes identified as hubs exclusively for a certain classification, we can check their rankings computed in the other classification. Commonly, these genes are placed in high positions (green and purple boxes in the vectors in Figure 3), suggesting that the hubs have a key role in the networks, independently from the considered classification. Only a few genes do not follow this trend (dotted arrows in Figure 3). In particular, in 2021 GBM we identified the *CCNA2* (Cyclin-A2) F2 in 2016 GBM. *KIAA1045* (PHD finger protein 24) and *SYT13* (Synaptotagmin 13) genes have been recognized as hubs in 2021 astrocytoma, but the average of their 2016 percentage measures is 4 and 8, respectively. A literature research on these genes revealed that all of them have been associated with glioma by different bioinformatic analyses,^34,35^ and *CCNA2* has been recently proposed as an immunologic biomarker for GBM.^36^

**Figure 4.**
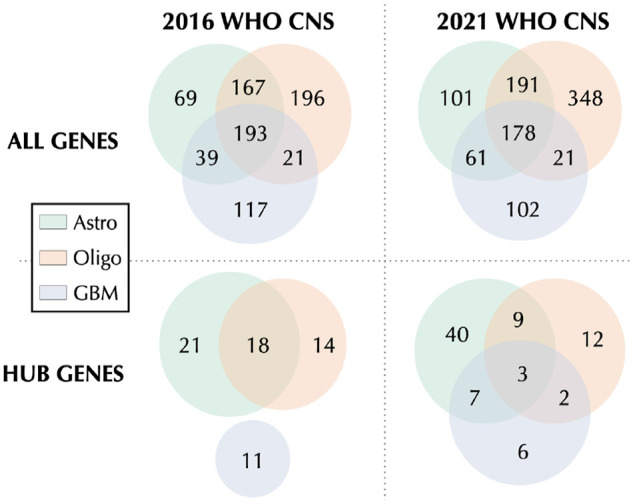
Venn diagrams comparing the variables selected by glasso from each glioma type (astrocytoma—Astro, oligodendroglioma—Oligo, and GBM). The first and second columns show glasso results, respectively, for 2016 and 2021 WHO CNS classifications. The first row considers all the selected genes, whereas the second row is only about hub genes.

As shown in Figure 4 (first row), for both classification procedures, the 3 tumor types share a considerable number of features. Nevertheless, we can observe more similarities in gene selection between astrocytoma and oligodendroglioma, compared with GBM. This result has been also confirmed by the analysis of the hub genes (Figure 4; second row), especially following the 2016 WHO CNS classification, where the set of GBM hubs does not intersect with the others. The comparison of these diagrams also shows a big difference in hub selection, in shared/exclusive genes, revealing the impact of the classification procedure in the selection of key features.

### Survival analysis

For each classification, survival analysis was performed by considering data sets comprising all the glioma samples; case 1 considers the complete set of variables selected by glasso, case 2 considers only the subset of variables exclusively selected from each glioma type, or case 3 considers only the identified hub genes. We should note that the data set construction depends on the class labels, as the variables constituting each data set have been selected by applying glasso algorithm on each glioma type, separately. However, the Cox regression model fitting followed an unsupervised approach, ie, the model learnt through data that was unlabelled. Since the glioma types often exhibit different prognosis, as validation of our results, we expect to find an association between the given diagnostic label and the prediction of survival.

**Figure 5.**
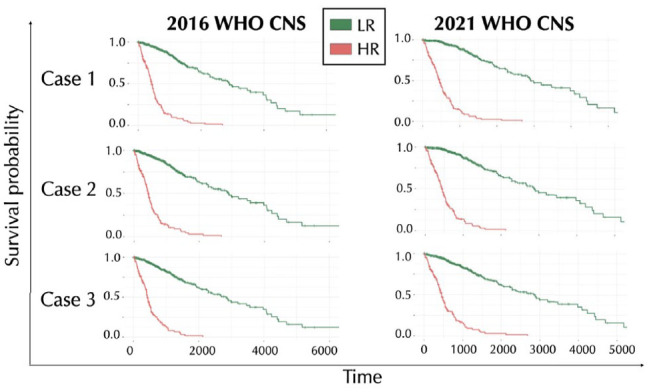
Kaplan-Meier survival curves obtained by the regularized Cox regression method applied to our cases of study. Case 1 considers a starting data set comprising all the selected variables. Case 2 refers to the variables exclusively selected from each glioma type. Case 3 considers only the hub genes. Green lines represent the survival probability of LR sample group, whereas red lines refer to the HR sample group.

Overall, our sets of variables allow a statistically significant separation (*P* ${<10}^{-16}$ ) of the 2 HR and LR groups, as shown in the Kaplan-Meier survival curves in Figure 5. We observe that, although case 2 considers a widely reduced data set than case 1 (Supplemental Table S2), the survival results are comparable. Moreover, the 2021 WHO CNS classification leads to better results than 2016, since, in every case, the 2 curves have a clearer separation.

To analyze the 2 HR and LR groups, we first compared the patient stratification obtained in the 3 cases. For both the classifications, most of the samples are recognized to be part of the same group, independently from the case of study, ie, about 90% and 78% of samples are stably in the LR and HR groups, respectively, even by changing the reference data set. As a consequence, to explore the tumor types associated with the samples constituting these 2 groups, we can focus on one of these cases without loss of generality. In particular, we discuss case 1, as its starting data set includes both the sets of variables of cases 2 and 3, increasing the reliability of the corresponding results.

Table 1 resumes the numbers and percentages of HR and LR samples identified in case 1, by specifying their glioma type.

**Table 1.** Cross-comparison between the composition of high- and low-risk groups and sample glioma types, depending on glioma classification.

Glioma type	2016 WHO CNS		2021 WHO CNS	
	High-risk	Low-risk	High-risk	Low-risk
	179 (28%)	464 (72%)	202 (33%)	417 (67%)
Astrocytoma	24 (13%)	246 (53%)	13 (6%)	241 (58%)
Oligodendroglioma	7 (4%)	217 (47%)	0 (0%)	166 (40%)
GBM	148 (83%)	1 ( <1 %)	189 (94%)	10 (2%)

Considering the 2016 variables, the HR group contains 179 samples. According to 2016 WHO CNS, 82.7% of them (n=148) are classified as GBM, whereas the remaining 17.3% are astrocytoma (n=24) and oligodendroglioma (n=7). Curiously, most of the LGG samples (astrocytoma and oligodendroglioma) being part of this group, in 2021 change their classification to GBM. Conversely, the 2016-LR group is constituted of 464 samples, among which 99.8% are LGG. Only 1 GBM sample is included in the 2016-LR group, which is diagnosed as astrocytoma in the 2021 classification. On the other side, the data set obtained from the 2021 selected variables leads to a HR group of 202 samples. The patients classified as GBM constitute 93.6% of them, whereas the other 6.4% are astrocytoma. There are no oligodendroglioma samples in the 2021-HR group. The LR group is mainly constituted by LGG (97.6%), but there are 10 GBM samples. If we look at the histological features reported by the TCGA clinical information, 5 more than 13 2021-astrocytoma samples being part of the HR group were histologically evaluated as GBM, as well as 9 more than 10 2021-GBM samples have histological features of LGG (5 astrocytoma, 2 oligodendroglioma, and 2 mixed).

The regularized Cox regression model allowed for the selection of relevant features to describe the patient’s survival data. In case 1, 24 and 23 genes are identified, respectively, from the 2016 and 2021 classification data sets. We observed that 50% of the 2016 genes were also selected either in case 2 (n=9) or case 3 (n=3). This percentage increases by considering the 2021 variables, reaching 74% (15 genes in case 2 and 2 genes in case 3). To investigate the role of these genes in glioma disease, we performed a literature review. Among the 35 genes, 12 are already known to have a role in glioma, whereas 13 are either linked to other cancer processes or pointed out in relation to glioma by bioinformatic studies. We did not find any cancer-related information for the remaining 10 genes. More details are summarized in Supplemental Table S3.

### Gene networks from 2021 WHO CNS classification

**Figure 6.**
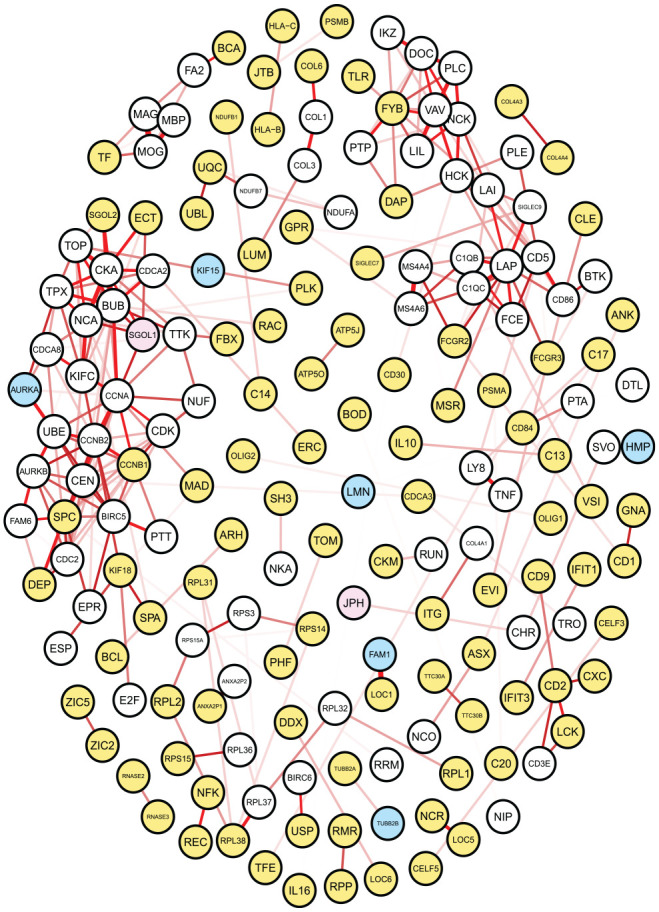
GBM gene network estimated through glasso. Nodes represent genes exclusively selected from GBM data set (yellow) and others directly linked with them (white). Blue and pink nodes highlight genes with prognostic value, according to regularized Cox model, which could in turn be exclusive for GBM or shared, respectively. Network layout stresses the adjacency between nodes, based on the Fruchterman-Reingold force-directed algorithm. Edge thickness depends on the strength of the corresponding connection.

The previous analyses denoted the 2021 WHO CNS classification as the most suitable for studying the glioma types. Moreover, survival analysis recognized the set of exclusive genes as very informative for prognosis, since, in 2021, they represent 74% of the variables identified as important to predict the patient’s survival. For this reason, in this section, we focus on the networks estimated through glasso on the 2021 data set, to further explore the relations linking the exclusive genes, which we expect better characterize the different glioma types. These networks can serve as a tool to detect genes of interest as potential novel biomarker. For example, Figure 6 shows the GBM exclusive network. It includes a subset of nodes composed of the genes exclusively selected for GBM (yellow), and the ones directly linked with them (white). Blue and pink nodes represent the genes selected as relevant in the survival analysis, which could be exclusive or shared by more than 1 tumor type, respectively. The chosen layout for graph representation is based on the Fruchterman-Reingold force-directed algorithm implemented in **qgraph** R package,^37^ which distributes nodes based on the distance between them: adjacent nodes are close, whereas not-connected nodes are apart.

This network comprises 175 nodes, among which 102 are genes exclusively selected from GBM data set. On the left side, we can observe a dense group of nodes, including genes selected for their prognostic value (*AURKA*—Aurora kinase A—and—*SGOL1*—Shugoshin 1). The network includes 6 exclusive genes selected by the regularized Cox regression model, and some of them are involved in very strong connections, represented by bold edges. Specifically, the 2 genes *HMP19* (Neuronal Vesicle Trafficking Associated 2) and *FAM115C* (TRPM8 Channel-Associated Factor 2) are predicted to be linked with *SVOP* (Synaptic Vesicle 2-Related Protein) and *LOC154761* (Family With Sequence Similarity 115, Member C Pseudogene), respectively. A strong correlation can be also observed between *AURKA* and *UBE2C* (Ubiquitin-conjugating Enzyme E2C).

This network representation has been reproduced also for the other 2 glioma types (Supplemental Figures S2 and S3). By the analysis of the astrocytoma network, we identified a potential interesting subnetwork constituted by 3 exclusive genes, *FAM123C* (APC Membrane Recruitment Protein 3)—also selected in the regularized survival model)—*ACTL6B* (Actin Like 6B), and *INA* (Internexin Neuronal Intermediate Filament Protein Alpha). Interestingly, a study about Melanoma Differentiation Associated gene - 9 (MDA-9)/Syntenin dysregulation in cancer pointed out these 3 genes as highly downregulated in glioma with high MDA-9/syntenin expression.^38^ Observing the oligodendroglioma network, we discovered that it contains all the exclusive genes that in our literature review ended with no information. One of them, RAB36, Member RAS Oncogene Family, appears strongly connected in biggest subnetwork, in which also appear many oligodendroglioma genes, such as TSPYL5 (Testis-specific Y-encoded-like protein 5), RASAL1 (Rat sarcoma (RAS) protein activator like 1), andKLHL26 (Kelch Like Family Member 26).

## Discussion

The main goal of this work was to discuss differences and similarities at the transcriptomic level between the 2 most recent glioma classifications, provided by the 2016 and 2021 WHO CNS guidelines. The glioma type of each sample of TCGA RNA-Seq data set was updated, and the glasso algorithm was applied to each glioma subgroup. The sets of selected variables were analyzed and used as the starting point for survival analysis performed with regularized Cox model and consequent patient stratification.

Our results suggest that the 2 classification procedures do not provide remarkable differences in variable selection. Even if the algorithm identified variables exclusively related to 1 classification, the corresponding rankings are low. Moreover, all the identified hubs are part of the subset of selected features, independently from the classification. However, comparing the hub subsets, our results highlight that the 2 procedures identified different key genes, suggesting that the classification could affect the detection of potential biomarkers specific to each glioma type. In particular, for GBM and astrocytoma, the 2021 classification provides larger sets of hub genes, including almost all the 2016 hubs. In addition, our analysis reveals 3 2021 hub genes, 1 from GBM and 2 from astrocytoma, having low rankings according to the 2016 classification. This seems to indicate that grouping the samples following the 2021 guidelines leads to a more comprehensive list of key features.

The statistically significant stratification of patients into HR and LR groups derived by survival analysis indicates that our selected sets of variables carry enough information to divide samples into HR and LR groups, serving also as validation of the glasso results.

Moreover, most of the variables selected as genes able to describe survival in case 1 (which took into account all the variables selected by glasso) have also been pointed out as output in case 2, in which the data set considered only variables exclusively selected by each glioma type. This outcome is particularly interesting, since while the genes commonly selected by all the glioma types could be either related to specific-glioma mechanisms or general cell functions, the exclusive genes can be considered more characteristic of the corresponding glioma type. In this light, these results suggest that variables that have been selected based on their relevance for each glioma type (diagnostic value) also hold prognostic information.

The patient stratification into HR and LR groups derived from survival model fitting is consistent with literature,^39^ since GBM samples are associated with the worst survival, and they constitute the major part of the HR group. The 2021 classification leads to better results in both distinction with statistical significance of LR and HR groups, and the association between GBM and poor survival, given that the percentage of GBM samples in the HR group increases by more than 10% compared with 2016 sample grouping.

As expected, the unsupervised sample grouping into HR and LR underlines the presence of some unconventional associations between glioma subtypes and overall survival. Based on the 2016 variable selection, the survival returns a HR group containing 179 samples. Despite 83% of them being classified as GBM, few LGG samples are predicted to have poor survival. If we classify them according to the new 2021 WHO CNS guidelines, we observe that 84% of these samples are diagnosed as GBM. Similarly, the only GBM sample being part of the 2016-LR group presents IDH mutation, but no 1p/19q codeletion, which turns out to be astrocytoma in 2021. Although this result seems to support the idea that the 2021 classification leads to more consistent survival outcomes, this does not occur. Indeed, even if the number of LGG in the HR group is lower than the one predicted by considering the 2016 classification, there are 10 GBM assigned to the LR group. Interestingly, we can assess that 9 out of 10 2021-GBM samples have histological features of LGG. This result is coherent with other studies that highlight the possibility of glioma samples presenting IDH mutation (which should be LGG in 2021) associated with poor outcomes, as well as IDH-wildtype (necessary condition to be GBM) with favorable survival.^26,19^ All these results suggest that there are still unknown factors influencing survival, which could also affect tumor histology.

The literature research on the 37 genes identified as having prognostic relevance revealed that 34% of them are known to have a role in glioma progression. Other 20% have been identified as key in other cancers, whereas 17% have been recognized as important by in silico analyses on glioma data sets. The remaining 29% features are not linked to cancer-related processes. The genes not previously investigated in glioma could be suitable for further investigation. Moreover, the fact that most of the genes identified in this study either arose from bioinformatic studies on glioma or are involved in cancer processes supports the necessity of validating them biologically.

The provided network visualization could be used as a tool to disclose unknown relations and to support biological research. Allowing for the detection of relevant subnetworks within glioma types, network inference offers valuable insights into glioma development mechanisms. For instance, our study uncovered a robust link between *LOC154761* and *FAM115C* in the GBM network. According to the performed multivariate Cox regression model, *FAM115C* emerged among the genes significantly contributing to the differentiation between HR and LR patients, a result that aligns with previous research identifying it as an oncogene implicated in promoting glioma malignancy and affecting prognosis.^40^ A recent study has also demonstrated that *FAM115C* overexpression promotes glioma cell migration and invasion,^41^ traits that characterize GBM progression. On the contrary, whereas the functional role of *LOC154761* in glioma disease remains unexplored, its downregulation in GBM compared with normal tissue^42^ suggests a possible involvement of this gene in cancer-related processes.

Another noteworthy result of our network analysis was the detection of a strong relation between *AURKA* and *UBE2C* genes, indicating their potential combined importance in GBM regulation. Prior studies on glioma have shown *AURKA*’s role in cancer cell self-renewal,^43^ whereas overexpression of *UBE2C* has been linked to aggressive tumor progression.^44^ Notably, research in colorectal cancer has demonstrated that the *AURKA*-mediated overexpression of *UBE2C* may produces a synergistic oncogenic effect, exacerbating tumor characteristics.^45^ Although the combined impact of *AURKA* and *UBE2C* in glioma remains underexplored, a previous study focused on the correlation between *UBE2C* and *AURKB* (Aurora kinase B),^46^ a gene sharing a similar role to *AURKA* in cell division during mitosis and tumorigenesis.^47^ This research highlights that the genes’ co-overexpression in glioma correlated with poor prognosis and therapy resistance, suggesting *AURKB* and *UBE2C* as potential therapeutic targets. These findings, and the high functional similarity between *AURKA* and *AURKB*, strongly support the potential involvement of the *AURKA* and *UBE2C* relationship in GBM pathogenesis. Further biological studies are warranted to elucidate the combined impact of these genes and explore their therapeutic implications.

## Conclusions

To our knowledge, this is the first work discussing differences and similarities between the 2 most recent glioma classifications in their impact on potential biomarker identification for each glioma type.

According to our results, transcriptomics data seem to align with the current changes in glioma classification guidelines, since the new 2021 WHO CNS classification provides better results in variable selection and identification of key features, compared with the previous version of 2016.

The survival analysis allows the separation of samples into the HR and LR groups, proving that the sets of variables we identified carry information also from the prognostic point of view. The 2021 classification led to better stratification of patients based on their survival profiles, since the percentage of GBM (characterized for worst prognosis than LGG) in the HR group increased compared with the 2016 classification, as expected. Moreover, with our pipeline, we provide a useful way to disclose unknown gene relations through network visualization.

Overall, our study brings insight into new features with a diagnostic and prognostic value that can be further biologically evaluated. However, the presence of unexpected associations between glioma types and predicted survival suggests that, although molecular features are essential in predicting patient survival, histology has an impact on the risk of death. Therefore, additional efforts are needed to further characterize the heterogeneity of glioma types and improve their overall classification. We hope our results motivate the scientific community to further investigate the role of the genes we identified, intending to improve glioma therapies.

## Supplemental Material

sj-pdf-1-bbi-10.1177_11779322241271535 – Supplemental material for Inferring Diagnostic and Prognostic Gene Expression Signatures Across WHO Glioma Classifications: A Network-Based Approach Supplemental material, sj-pdf-1-bbi-10.1177_11779322241271535 for Inferring Diagnostic and Prognostic Gene Expression Signatures Across WHO Glioma Classifications: A Network-Based Approach by Roberta Coletti, Mónica Leiria de Mendonça, Susana Vinga and Marta B. Lopes in Bioinformatics and Biology Insights
