# Supplementary material for: Inferring Diagnostic and Prognostic Gene Expression Signatures Across WHO Glioma Classifications: A Network-Based Approach
Source: Bioinform Biol Insights. 2024 Sep 15;18:11779322241271535. doi: 10.1177/11779322241271535 (PMC11403688; doi:10.1177/11779322241271535)
Supplement: sj-pdf-1-bbi-10.1177_11779322241271535 – Supplemental material for Inferring Diagnostic and Prognostic Gene Expression Signatures Across WHO Glioma Classifications: A Network-Based Approach [file sj-pdf-1-bbi-10.1177_11779322241271535.pdf]

Supplementary Material

S1. Mathematical validation

Mathematical validation of variable selection derived from glasso results has been performed to appraise the consistency of the solution of the equation (1). Let D be the RNASeq dataset constituted by the subset of selected variables for a given glioma type. Setting a very low value of the regularization parameter ρ, we can determine a matrix Θ_D that does not lead to further variable selection[1]. Considering the objective function as a difference between two functions of Θ, i.e. $F(\Theta) - R(\Theta)$, where $F(\Theta) = \log(\det\Theta) - tr(S\Theta)$, and $R(\Theta) = \rho||\Theta||_1$, we compared the values of F obtained from the dataset D (F_D), with the one computed from 1000 random variable selections of the same dimension (F_R). If $F_D > F_R$ for all 1000 random datasets, we can conclude that the set of selected variables really led to the optimal solution. The results of the mathematical validation performed for both glioma classifications and for each glioma type are reported in Table 1, together with the fixed regularization parameters.

We noticed that, although the optimization results depend on the regularization term $R(\Theta)$, choosing low values of ρ we obtain $O(F(\Theta)) \gg O(R(\Theta))$, such that the regularization term can be ignored.

[1]The value of ρ should be different for any glioma-type, since everyone led to a different set of variable selected, determining different dimension of the dataset D.

	2016 WHO CNS								
	ρ	F_D	F_R Best	F_R Average	F_R Median	R_D	R_R Best	R_R Average	R_R Median
Astro	0.05	476.12	76.69	52.93	53.24	0.79	0.62	0.64	0.63
Oligo	0.08	496.62	76.58	50.65	50.69	0.85	0.74	0.73	0.73
GBM	0.02	490.21	239.8590	225.44	225.44	0.68	0.57	0.57	0.57
	**2021 WHO CNS**								
Astro	0.08	453.46	10.68	-9.69	-9.83	0.86	0.74	0.73	0.73
Oligo	0.13	467.82	-6.27	-32.43	-32.17	0.90	0.86	0.83	0.83
GBM	0.05	347.33	51.09	34.36	34.15	0.75	0.61	0.61	0.61

Table 1: Mathematical validation results. For each WHO CNS classification, the values of $F(\Theta)$ and $R(\Theta)$ functions are reported. F_D and R_D are computed by considering the dataset with the selected variables only. F_R and R_R summarize the results obtained for random variable subsets by showing the best, the average and median values computed. Table also shows in the first column the values of the regularization parameter ρ fixed in each case. Astro: astrocytoma; Oligo: oligodendroglioma; GBM: glioblastoma.

S2. Additional figures and tables

**WHO CNS**	**Case**	**Dataset dimension** (n x p)	λ
2016	1	643 x 802	0.062
	2	643 x 382	0.059
	3	643 x 64	0.010
2021	1	619 x 1002	0.059
	2	619 x 551	0.054
	3	619 x 79	0.014

Table 2: For each case, dataset dimension and parameters setting are reported. Case 1 considers a starting dataset comprising all the selected variables. Case 2 refers to the variables exclusively selected from each glioma subtype. Case 3 considers only the hub genes. *pmax* has been computed by dividing the number of dead samples by 10 (EPV). λ is the Lasso regularization parameter.

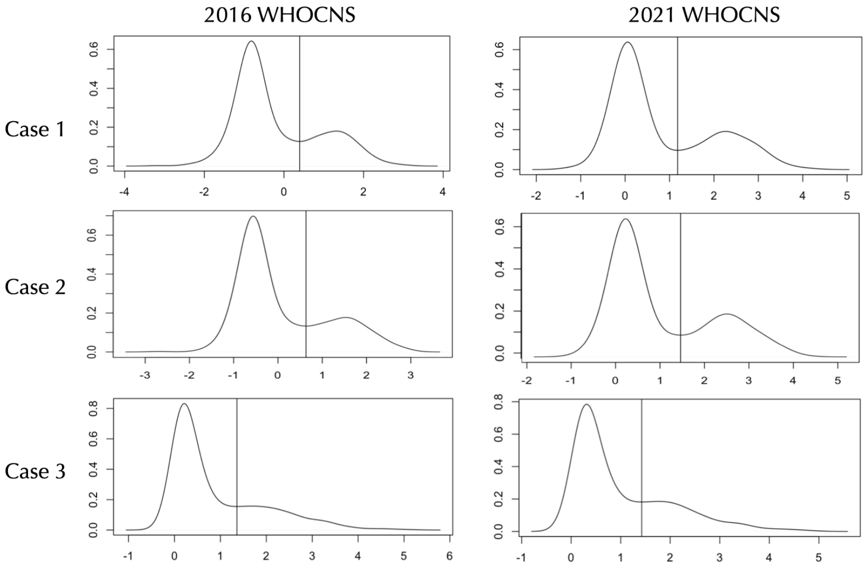

Figure S1: Density functions of the prognostic indexes (PI) of the samples constituting the dataset in each case. Case 1 considers a starting dataset comprising all the selected variables. Case 2 refers to the variables exclusively selected from each glioma subtype. Case 3 considers only the hub genes. The vertical lines represent the chosen thresholds $\hat{PI}$ to divide samples into high- and low-risk groups. Samples with PI $\leq \hat{PI}$ were assigned to the low-risk group, conversely, they are assigned to the high-risk group.

**Gene**	**WHO CNS**	**Knowledge**	**Ref.**
AGAP4	2016	–	–
HMP19	2016	–	–
USP34	2016	Downregulation inhibits pancreatic cancer growth and migration	[1]
SAMD8	2016	–	–
TMEM176A	2016	Promoter of GBM cell growth.	[2]
MYBL2	2016	Overexpression induces esophageal squamous-cell carcinoma proliferation	[3]
OSCAR	2016	Bioinformatic analysis reveled the expression of this gene correlates with poor prognosis in multiple cancer (including glioma)	[4]

RRM2	2016	It contributes to the migration and proliferation of glioma	[5]
CLEC18B	2016	Overexpression induces proliferation, migration, and bad prognosis of GBM	[6]
PABPC3	2016	Possible gene associated with predisposition to breast cancer in North African population	[7]
CDC6	2016	Upregulated in glioma. Correlated with immune infiltrates and poor survival.	[8, 9]
CLEC18A	2016	–	–
WAPAL	2021	–	–
RPL13A	2021	Bioinformatic analysis revealed it is deferentially expressed in glioma.	[10]
JPH3	2021	Tumor suppressor in hepatocellular carcinoma.	[11]
TUBB2B	2021	Related to GBM invasion	[12]
HJURP	2021	Involved in GBM proliferation and radioresistance. Terapeutic target.	[13]
CPPED1	2021	Prevent bladder cancer progression.	[14]
ARHGAP11A	2021	High expression correlates with better prognosis in gastric cancer. It determines malignant progression of hepatocellular carcinoma	[15, 16]
RAB36	2021	–	–
POC1A	2021	Bioinformatic analysis revealed it is highly expressed in multiple cancer (including glioma)	[17]
LRRC61	2021	GBM prognostic marker according to multi-omics bioinformatic analysis.	[18]
PGCP	2021	–	–
DHRS4	Both	Gene knockdown reduces glioma proliferation, invasion, and migration.	[19]
EEF1A1P9	Both	Bioinformatic analysis revealed that this pseudogene predicts survival in glioma	[20]

RPL7A	Both	Bioinformatic analysis revealed that it is upregulated and related to survival in GBM.	[21]
SNAP91	Both	Downregulated and correlated with survival in GBM.	[22]
EIF3L	Both	–	–
POM121C	Both	–	–
GTF2IRD2	Both	Role in the neurodevelopmental disorder Williams-Beuren syndrome.	[23]
S100A11	Both	Its expression positively correlates with glioma survival.	[24]
FAM115C	Both	Tumor suppressor associated with prolonged survival in pancreatic cancer.	[25]
KIF20A	Both	Overexpression promotes glioma progression. Downregulation inhibits glioma tumorigenesis.	[26, 27]
AURKA	Both	Involved in the self-renewal of in glioma-initiating cells.	[28]
SGOL1	Both	Bioinformatic analysis revealed that downregulation determines better prognosis in glioma.	[29]

Table 3: Literature research has been performed on genes identified by the regularized Cox regression algorithm to have predictive significance. The second column specifies if the dataset leading to the gene selection has been created by considering 2016 or 2021 classifications (or both). If the research resulted in noteworthy information, they are reported with the corresponding reference.

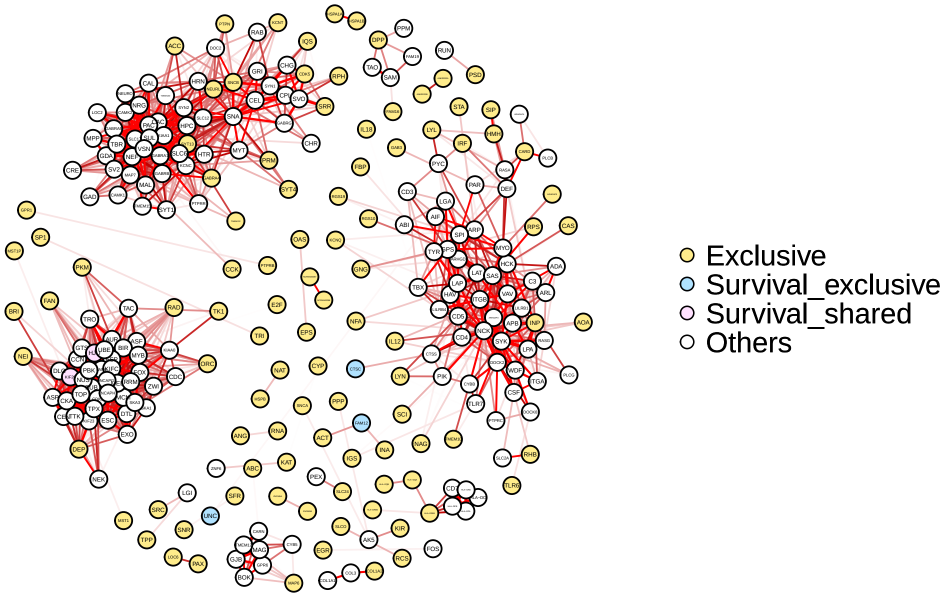

Figure S2: Astrocytoma gene-network estimated through glasso. Nodes represent genes exclusively selected from astrocytoma dataset (yellow) and others directly linked with them (white). Blue and pink nodes highlight genes with prognostic value, according to regularized Cox model, which could in turn be exclusive for astrocytoma or shared, respectively. Network layout stresses the adjacency between nodes, based on the Fruchterman-Reingold force-directed algorithm. Edge thickness depends on the strength of the corresponding connection.

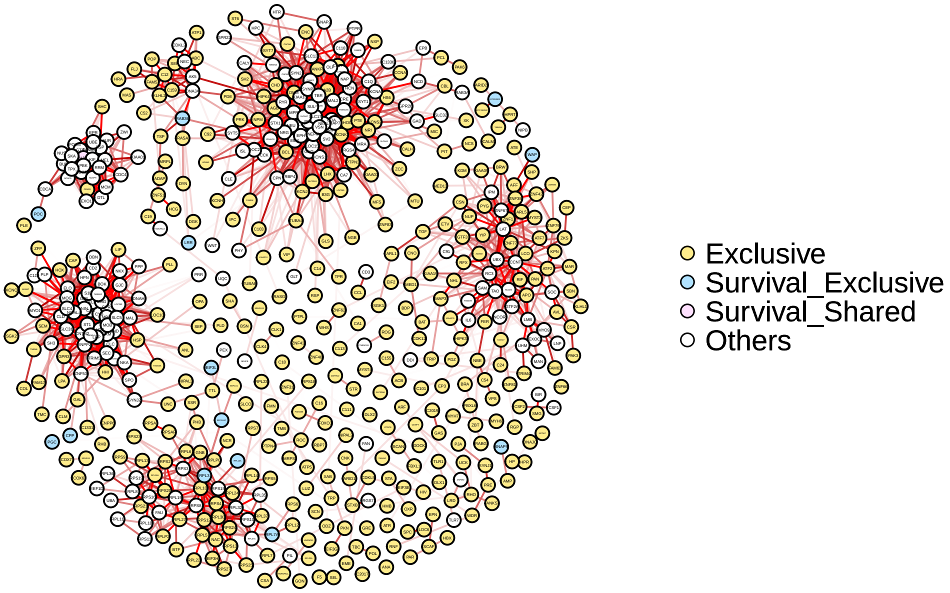

Figure S3: Oligodendroglioma gene-network estimated through glasso. Nodes represent genes exclusively selected from oligodendroglioma dataset (yellow) and others directly linked with them (white). Blue and pink nodes highlight genes with prognostic value, according to regularized Cox model, which could in turn, be exclusive for oligodendroglioma or shared, respectively. Network layout stresses the adjacency between nodes, based on the Fruchterman-Reingold force-directed algorithm. Edge thickness depends on the strength of the corresponding connection.

References

[1] C. Lin, J. Xia, Z. Gu, Y. Meng, D. Gao, S. Wei, Downregulation of usp34 inhibits the growth and migration of pancreatic cancer cells via inhibiting the prr11, OncoTargets and Therapy 13 (2020) 1471–1480. doi:10.2147/OTT.S228857.

[2] Z. Liu, H. An, P. Song, D. Wang, S. Li, K. Chen, Q. Pang, Potential targets of tmem176a in the growth of glioblastoma cells, OncoTargets and Therapy 11 (2018) 7763–7775. doi:10.2147/OTT.S179725.

[3] H. Qin, Y. Li, H. Zhang, F. Wang, H. He, X. Bai, S. Li, Prognostic im-

plications and oncogenic roles of mybl2 protein expression in esophageal squamous-cell carcinoma, OncoTargets and Therapy 12 (2019) 1917–1927. doi:10.2147/OTT.S190145.

[4] X. Liao, Y. Bu, Y. Zhang, B. Xu, J. Liang, Q. Jia, C. Zhang, Oscar facilitates malignancy with enhanced metastasis correlating to inhibitory immune microenvironment in multiple cancer types, Journal of Cancer 12 (2021) 3769–3780. doi:10.7150/jca.51964.

[5] H. Sun, B. Yang, H. Zhang, J. Song, Y. Zhang, J. Xing, Z. Yang, C. Wei, T. Xu, Z. Yu, Z. Xu, M. Hou, M. Ji, Y. Zhang, Rrm2 is a potential prognostic biomarker with functional significance in glioma, International Journal of Biological Sciences 15 (2019) 533–543. doi:10.7150/ijbs.30114.

[6] R.-M. Guo, C.-B. Zhao, P. Li, L. Zhang, S.-H. Zang, B. Yang, Overexpression of clec18b associates with the proliferation, migration, and prognosis of glioblastoma, ASN Neuro 10 (2018) 1759091418781949. doi:10.1177/1759091418781949.

[7] Y. Hamdi, M. Boujemaa, M. B. Rekaya, C. B. Hamda, N. Mighri, H. E. Benna, N. Mejri, S. Labidi, N. Daoud, C. Naouali, O. Messaoud, M. Chargui, K. Ghedira, M. S. Boubaker, R. Mrad, H. Boussen, S. Abdelhak, the P E C Consortium, Family specific genetic predisposition to breast cancer: results from tunisian whole exome sequenced breast cancer cases, Journal of Translational Medicine 16 (2018) 158. doi:10.1186/s12967-018-1504-9.

[8] H. Zhao, X. Zhou, G. Yuan, Z. Hou, H. Sun, N. Zhai, B. Huang, X. Li, Cdc6 is up-regulated and a poor prognostic signature in glioblastoma multiforme, Clinical and Translational Oncology 23 (2021) 565–571. doi:10.1007/s12094-020-02449-w.

[9] F. Wang, F. Zhao, L. Zhang, L. Xiong, Q. Mao, Y. Liu, X. Qiu, X. Wang, L. Shui, X. Chen, K. Ren, P. Shui, Q. Zhang, Y. Deng, W. Li, X. Xie, D. Wu, T. Li, J. Lang, L. Liu, H. Chen, J. Xu, S. Bai, Z. Li, Q. Yue, N. Chen, B. Zhou, C. Yi, Y. Wei, Y. Fu, Y. Luo, Q. Gou, L. Liu, Y. Liu, J. Kang, J. Wang, D. Jing, F. Zhang, X. Yang, X. Li, T. Jiang, Z. Zhang, Y. Zhou, J. Yi, Cdc6 is a prognostic biomarker and correlated with immune infiltrates in glioma, Molecular Cancer 21 (2022) 153. doi:10.1186/s12943-022-01623-8.

[10] S. Kreth, J. Heyn, S. Grau, H. A. Kretzschmar, R. Egensperger, F. W. Kreth, Identification of valid endogenous control genes for determining gene expression in human glioma, Neuro-Oncology 12 (2010) 570–579. doi:10.1093/neuonc/nop072.

[11] Y. Huang, Z. Yu, M. Zheng, X. Yang, H. Huang, L. Zhao, Methylation-associated inactivation of jph3 and its effect on prognosis and cell biological function in hcc, Molecular Medicine Reports 25 (4 2022). doi:10.3892/mmr.2022.12640.

[12] E. Yeini, P. Ofek, S. Pozzi, N. Albeck, D. Ben-Shushan, G. Tiram, S. Golan, R. Kleiner, R. Sheinin, S. I. Dangoor, S. Reich-Zeliger, R. Grossman, Z. Ram, H. Brem, T. M. Hyde, P. Magod, D. Friedmann-Morvinski, A. Madi, R. Satchi-Fainaro, P-selectin axis plays a key role in microglia immunophenotype and glioblastoma progression, Nature Communications 12 (2021) 1912. doi:10.1038/s41467-021-22186-0.

[13] R. B. Serafim, C. Cardoso, L. F. M. D. Cristofaro, C. P. Soares, W. A. Silva, E. M. Espreafico, M. L. Paçó-Larson, B. D. Price, V. Valente, Hjurp knockdown disrupts clonogenic capacity and increases radiation-induced cell death of glioblastoma cells, Cancer Gene Therapy 27 (2020) 319–329. doi:10.1038/s41417-019-0103-0.

[14] D.-X. Zhuo, X.-W. Zhang, B. Jin, Z. Zhang, B.-S. Xie, C.-L. Wu, K. Gong, Z.-B. Mao, Cstp1, a novel protein phosphatase, blocks cell cycle, promotes cell apoptosis, and suppresses tumor growth of bladder cancer by directly dephosphorylating akt at ser473 site, PLOS ONE 8 (2013) e65679–.

[15] B. Fan, K. Ji, Z. Bu, J. Zhang, H. Yang, J. Li, X. Wu, Arhgap11a is a prognostic biomarker and correlated with immune infiltrates in gastric cancer, Frontiers in Molecular Biosciences 8 (2021).

[16] B. Dai, X. Zhang, R. Shang, J. Wang, X. Yang, H. Zhang, Q. Liu, D. Wang, L. Wang, K. Dou, Blockade of arhgap11a reverses malignant progress via inactivating rac1b in hepatocellular carcinoma, Cell Communication and Signaling 16 (2018) 99. doi:10.1186/s12964-018-0312-4.

[17] Q. Zhao, S. Gao, X. Chen, X. Zhu, Poc1a, prognostic biomarker of immunosuppressive microenvironment in cancer, Aging 14 (2022) 5195–5210. doi:10.18632/aging.204141.

[18] C.-G. Lei, X. Jia, W.-J. Sun, Establish six-gene prognostic model for glioblastoma based on multi-omics data of tcga database., Yi chuan = Hereditas 43 7 (2021) 665–679.

[19] Y. Dai, Z. Chen, W. Zhao, G. Cai, Z. Wang, X. Wang, H. Hu, Y. Zhang, mir-29a-5p regulates the proliferation, invasion, and migration of gliomas by targeting dhrs4, Frontiers in Oncology 10 (2020).

[20] Y. Wang, X. Liu, G. Guan, Z. Xiao, W. Zhao, M. Zhuang, Identification of a five-pseudogene signature for predicting survival and its cerna network in glioma, Frontiers in Oncology 9 (2019).

[21] Z. Wang, X. Guo, L. Gao, Y. Wang, W. Ma, B. Xing, Glioblastoma cell differentiation trajectory predicts the immunotherapy response and overall survival of patients, Aging 12 (2020) 18297–18321. doi:10.18632/aging.103695.

[22] Y.-F. Gao, X.-Y. Mao, T. Zhu, C.-X. Mao, Z.-X. Liu, Z.-B. Wang, L. Li, X. Li, J.-Y. Yin, W. Zhang, H.-H. Zhou, Z.-Q. Liu, Col3a1 and snap91: novel glioblastoma markers with diagnostic and prognostic value, Oncotarget 7 (2016).

[23] M. A. Porter, C. Dobson-Stone, J. B. J. Kwok, P. R. Schofield, W. Beckett, M. Tassabehji, A role for transcription factor gtf2ird2 in executive function in williams-beuren syndrome, PLOS ONE 7 (2012) e47457–.

[24] H. Wang, M. Yin, L. Ye, P. Gao, X. Mao, X. Tian, Z. Xu, X. Dai, H. Cheng, S100a11 promotes glioma cell proliferation and predicts grade-correlated unfavorable prognosis, Technology in Cancer Research and Treatment 20 (2021) 15330338211011961. doi:10.1177/15330338211011961.

[25] K. Saeki, H. Onishi, S. Koga, S. Ichimiya, K. Nakayama, Y. Oyama, M. Kawamoto, K. Sakihama, T. Yamamoto, R. Matsuda, Y. Miyasaka, M. Nakamura, Y. Oda, Fam115c could be a novel tumor suppressor associated with prolonged survival in pancreatic cancer patients, Journal of Cancer 11 (2020) 2289–2302. doi:10.7150/jca.38399.

[26] M. Wang, K. Liu, X.-L. Zhou, S.-Y. Mei, C.-J. Zhang, T.-G. Zhang, Downregulation of kif20a induces cell cycle arrest and apoptosis by sup-

pressing pi3k/akt in human glioblastoma, Int J Clin Exp Med 10 (2017) 16133–16143.

[27] J. Duan, W. Huang, H. Shi, Positive expression of kif20a indicates poor prognosis of glioma patients, OncoTargets and Therapy 9 (2016) 6741–6749. doi:10.2147/OTT.S115974.

[28] Z. Xia, P. Wei, H. Zhang, Z. Ding, L. Yang, Z. Huang, N. Zhang, Aurka governs self-renewal capacity in glioma-initiating cells via stabilization/activation of β-catenin/wnt signaling, Molecular Cancer Research 11 (2013) 1101–1111. doi:10.1158/1541-7786.MCR-13-0044.

[29] J. Dai, Z. Bing, Y. Zhang, Q. Li, L. Niu, W. Liang, G. Yuan, L. Duan, H. Yin, Y. Pan, Integrated mrnaseq and micrornaseq data analysis for grade iii gliomas, Molecular Medicine Reports 16 (2017) 7468–7478. doi:10.3892/mmr.2017.7545.
